# On the Analysis of Inter-Relationship between Auto-Scaling Policy and QoS of FaaS Workloads

**DOI:** 10.3390/s24123774

**Published:** 2024-06-10

**Authors:** Sara Hong, Yeeun Kim, Jaehyun Nam, Seongmin Kim

**Affiliations:** 1 Department of Convergence Security Engineering, Sungshin Women’s University, 2, Bomun-ro 34da-gil, Seongbuk-gu, Seoul 02844, Republic of Korea; sarahong970520@gmail.com (S.H.); yehihello119@gmail.com (Y.K.); 2 Department of Computer Engineering, Dankook University, 152, Jukjeon-ro, Suji-gu, Yongin-si 16890, Republic of Korea; jaehyun.nam@dankook.ac.kr

**Keywords:** Function-as-a-Service, auto-scaling, container, Kubernetes

## Abstract

A recent development in cloud computing has introduced serverless technology, enabling the convenient and flexible management of cloud-native applications. Typically, the Function-as-a-Service (FaaS) solutions rely on serverless backend solutions, such as Kubernetes (K8s) and Knative, to leverage the advantages of resource management for underlying containerized contexts, including auto-scaling and pod scheduling. To take the advantages, recent cloud service providers also deploy self-hosted serverless services by facilitating their on-premise hosted FaaS platforms rather than relying on commercial public cloud offerings. However, the lack of standardized guidelines on K8s abstraction to fairly schedule and allocate resources on auto-scaling configuration options for such on-premise hosting environment in serverless computing poses challenges in meeting the service level objectives (SLOs) of diverse workloads. This study fills this gap by exploring the relationship between auto-scaling behavior and the performance of FaaS workloads depending on scaling-related configurations in K8s. Based on comprehensive measurement studies, we derived the logic as to which workload should be applied and with what type of scaling configurations, such as base metric, threshold to maximize the difference in latency SLO, and number of responses. Additionally, we propose a methodology to assess the scaling efficiency of the related K8s configurations regarding the quality of service (QoS) of FaaS workloads.

## 1. Introduction

A recent innovation in serverless computing enables ease and flexible management of cloud-native applications. In contrast to legacy infrastructure-based service models, a client solely delegates the infrastructure management, including resource allocation and tenant isolation, to a service provider. Such a deployment model gives opportunities for the developer to concentrate more on the core logic of computerized services. Among the various paradigms of utilizing serverless architecture, Function-as-a-Service (FaaS) is a promising way to manage and run microservices cost-effectively and in a more fine-grained manner. The execution unit of FaaS is a stateless modular piece of code called a function that allows clients to run their services by triggering events with HTTP-based API requests. The FaaS platform takes the role of container orchestration with auto-scalability and multi-tenancy but is also responsible for application installation and configuration. To take advantage of the FaaS model, the global market leaders pioneered the trend by releasing commodity FaaS-based solutions, such as AWS Lambda [1], Microsoft Azure Functions [2], Google Cloud Functions [3], and IBM Cloud Functions [4].

In contrast to the pioneered FaaS solutions as public cloud offerings, recent service providers have utilized self-hosted serverless services to take advantage of its elasticity for a sensible usage of containerized resources [5]. When constructing self-hosted serverless services, the auto-scale subsystems and load balancers provided by Kubernetes and Knative runtime relieve the burden of managing dynamical loads adaptively. Such on-premise hosted platforms are typically implemented based on existing open-source FaaS platforms, such as Openwhisk, OpenFaaS, and Fission, and they heavily rely on Kubernetes (K8s) abstraction for hosting FaaS workloads, providing container orchestration and runtime environments [6,7,8]. Likewise, the commodity FaaS solutions also support composing serverless architecture on Kubernetes (e.g., Amazon EKS [9] and Azure AKS [10]) for container orchestration [11,12]. Besides, the Knative runtime, which runs serverless workloads on the Kubernetes cluster [11,13], unrolls private FaaS platforms on any cloud service providers (CSPs) if they set up Kubernetes-native environments. Such flexibility enables enterprises to utilize the FaaS platforms based on these runtimes to deal with on-premise data flows in cloud environments [14].

The horizontal pod auto-scaling (HPA), a default auto-scaling mechanism in Kubernetes, updates the number of deployment units, called pods, to automatically scale the workload to match demand and capacity dynamically [15]. To avoid a cold start that incurs additional latency associated with the startup time on a new pod when deploying workloads, HPA promptly scales out pods by monitoring the resource utilization (e.g., CPU by default) and gradually scales in the replicas [16]. Meanwhile, an overestimated profiling on resource utilization might cause excessive pod scaling, resulting in performance degradation of workloads due to the resource contention of the host, which, in turn, leads to a failure to meet the service level agreement (SLA) requirements. To address this, recent studies have focused on improving the efficiency of HPA by co-utilizing the vertical scaling in a hybrid manner [17] or leveraging the state-of-the-art reinforcement learning techniques to precisely forecast the resource utilization tendency [18,19,20]. Such efforts to deliver better performance of serverless applications have concentrated on improving the auto-scaler itself by (1) modifying the workflow of the auto-scaler for better scheduling or (2) changing the monitoring components to profile the resource utilization more precisely.

However, the lack of guidelines and recommendations for effective auto-scaling strategies makes accommodating proper decision-making to achieve QoS requirements challenging when building a self-hosted FaaS service. In the conventional FaaS model, FaaS clients often resort to FaaS providers (e.g., CSPs) to deal with configurations of the auto-scaler related to the application performance, including a default resource metric (e.g., CPU or concurrency) and target resource utilization to initiate the scale-out process. For the case of the existing FaaS solutions serviced by major CSPs, they offer various configurable options, such as initial resource allocation before launching containerized pods, for flexible provisioning [1,2,3]. Unfortunately, the corresponding performance metrics regarding auto-scaling behavior are abstracted due to the serverless nature of FaaS platforms [21]. Moreover, customer requirements vary across workload characteristics and user-perceived billing constraints. Similar to the case of such public cloud offerings, a service provider should determine the scaling-related configuration without explicit knowledge of auto-scaling tendency depending on the configurations. Such non-trivial design choices become challenges when deploying a private FaaS platform for enterprises responsible for determining the customizable metrics to meet the service-level-objective (SLO) requirements.

To this end, this paper explores a comprehensive empirical analysis of the state-of-the-art serverless auto-scalers when deploying and running FaaS workloads. To understand the dependency between configurable scaling-related metrics and the workloads’ performance, we conduct a diverse set of experiments on Kubernetes, a representative container orchestration framework. In addition, we thoroughly characterize the behavior of the default auto-scaler provided by Kubernetes (HPA) and Knative (KPA), providing guidelines for determining which method is beneficial depending on the conditions. To quantify the SLO-related performance and resource contention, we define a metric to assess the scaling efficiency and evaluate them while varying the customizable configurations related to auto-scaling. We believe that our study helps FaaS clients improve their workload performance and offers guidelines for building self-hosted FaaS solutions to improve resource efficiency and encompass the SLO requirements. In summary, our study makes the following contributions:

- Our study explores the configurable options of the state-of-the-art auto-scalers related to the performance of FaaS workloads and provides implications on setting the default metric, initial resource allocation, and scaling threshold.
- We define an evaluation metric for quantifying the scaling efficiency and methodology to evaluate the client’s QoS and SLO requirements for serverless computing workloads.
- Based on the comparison between the auto-scaling performance of HPA and KPA depending on the concurrency level of incoming requests, we provide a guideline for selecting a default auto-scaling mechanism.

The remainder of this paper will be organized as follows: Section 2 discusses the existing works regarding Kubernetes and Knative’s auto-scaling mechanisms. Section 3 describes our goal and analyzes the scaling metrics related to auto-scaling when using FaaS benchmarks. It also demonstrates what kind of benchmarks we used. Section 4 derives lessons on the auto-scaling efficiency of HPA from the result of diverse workloads depending on the custom metrics we discuss in Section 3. Section 5 gives helpful implications on choosing HPA or KPA and illustrates the process for a pod to become ready in Knative. Finally, we conclude our study in Section 6.

## 2. Background and Related Work

This section reviews the popular and representative auto-scaling mechanisms of state-of-the-art container orchestration and runtime environments. Then, we investigate the previous studies that have attempted to improve the existing auto-scalers and discuss the limitations and how their approaches differ from this study.

### 2.1. Auto-Scaling Mechanism of Kubernetes

Existing FaaS platforms utilize auto-scale subsystems provided by Kubernetes [22] container orchestration to deal with sudden overload and failure [14]. Kubernetes’ default auto-scaling mechanism is the horizontal pod auto-scaler (HPA). HPA updates a workload resource, called pods, to automatically scale out the workload to approximate the demand with computing resources for handling incoming requests [15]. Once incoming requests are loaded, the auto-scaler lets the controller manager daemon compare the collected metrics with the configured thresholds. When the average value of the metrics—such as CPU or memory utilization—reaches a predetermined threshold, HPA automatically scales out the number of replicas by calculating the following equation: *desiredReplicas* = [currentReplicas×currentMetricValue/desiredMetricValue] [16]. Several studies [16,23,24] have investigated the operational behaviors of HPA in Kubernetes and explored the factor (e.g., default metrics vs. custom metrics) that affects the auto-scaling performance. Manner et al. [14] implemented resource scaling strategies to compare and measure the performance of public and open-source FaaS platforms from the observation that all open-source serverless platforms rely on Kubernetes deployment.

In contrast to HPA, the vertical pod auto-scaler (VPA) controls the pods’ allocated resources rather than varying the number of pods by rescheduling the pod with the newly estimated resources [20]. As the scaling strategy aligns with the actual usage, it is known to manage resource utilization efficiently when the load of workloads dynamically changes. However, VPA might suffer from over-estimation when profiling the peak usage or disrupt service consistency as it requires eviction of existing pods or restarting them to adopt new policy [24]. To address this, RUBAS [20] leverages container migration and checkpointing to avoid over-estimation of resources and reduce the restart overhead, respectively.

The primary research directions have suggested improving the efficiency of the aforementioned auto-scaling mechanisms in two perspectives: (1) a profiling-based approach and (2) applying reinforcement learning. First, Libra [17], a hybrid approach that jointly manages horizontal and vertical scaling, was proposed to adaptively control the allocated resources and the number of pods depending on the load conditions. Libra controls the number of pods based on the HPA but vertically updates the resource limit of each pod when the load reaches the threshold. Ruíz et al. [25] presented a resource monitoring service to improve resource utilization and Quality of Service (QoS) by dynamically updating the Kubernetes auto-scaler’s behavior based on the SLO requirements. Similarly, Eismann et al. [26] proposed an approach to predict the optimal resource sizing of a serverless function using monitoring data from the last resource management task automatically.

Recent studies [18,19,20,27] have employed reinforcement learning techniques to scale out container-based applications adaptively. To accomplish the efficient horizontal and vertical elasticity of container-based serverless applications, G. Rattihalli et al. [20] explored the efficiency of three RL-based algorithms: Q-learning, Dyna-Q, and model-based RL. By exploiting different levels of algorithms depending on the knowledge of system dynamics adaptively, the authors integrated the proposed policies with Docker Swarm, and their simulation results show that the model-based RL policies deliver the most beneficial technique for scaling adaptation when deploying container-based applications at runtime. Fabiana Rossi et al. [27] also proposed RL-based policies that introduce self-adaptation capabilities in Docker Swarm, and Zhe Yang et al. [28] developed an adaptive resource allocation approach for a microservice workflow system using reinforcement learning. With their proposal, one can obtain an effective policy for resource allocation without prior knowledge of the microservice workflow system and simulator of the underlying system.

Schuler et al. [18] proposed an AI model based on reinforcement learning to optimize the concurrency level adaptively without prior knowledge of incoming workloads. They argued that the predefined concurrency level of an auto-scaler can strongly influence the performance of a serverless application on Knative. Similarly, the authors of [19] leveraged Q-learning to establish an adaptive auto-scaling for delay-sensitive serverless services in order to maintain the balance between resource efficiency and QoS. They demonstrated that the proposed approach accurately characterizes the workloads’ resource profile, and improves QoS and resource utilization compared to two existing state-of-the-art methods: KPA and Libra.

### 2.2. Knative Runtime and Its Auto-Scaler

**Figure 1:**
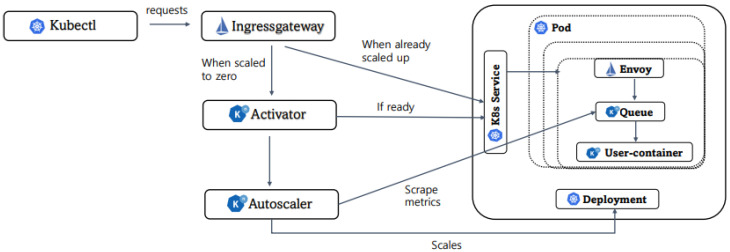
Typical FaaS architecture and system components utilizing Knative on Kubernetes.

Meanwhile, one of the mainstream trends in recent commodity FaaS platforms is to utilize the Knative runtime environment on them to be K8s-native [1,13]. Knative [29] is a third-party extension package for Kubernetes that offers primitives to deploy, manage, execute, and scale in/out functions while supporting multiple networking layers (e.g., Istio [30] and Contour [31]) and programming languages, including Node.js and Python v1.11.0. Figure 1 illustrates a typical FaaS deployment model that utilizes Knative on Kubernetes. The system components can be categorized into three parts: (1) K8s components, (2) Knative components, and (3) Istio components. The basic workflow is as follows: All incoming requests go through the envoy proxy from Istio, the ingress gateway. When there are not enough pods, the ingress gateway forwards the incoming requests to the activator. Then, it informs the load to the auto-scaler, and the auto-scaler scrapes metrics from the queue proxy to determine the number of replicas to be generated depending on the default policy (e.g., CPU utilization for HPA). Once ready, the activator notifies the readiness of pods to the K8s service, and it allocates and schedules the replicas. If scaled-out replicas are enough to handle the load, it forwards requests straight to the pod [18].

Knative pod auto-scaler (KPA) is a default auto-scaling policy in the Knative runtime. In contrast to Kubernetes HPA, KPA scales replicas based on concurrency, the average inbound requests per pod. KPA is beneficial for scale-to-zero functionality that allows pods to be run only when there is demand, enabling workloads with inbound synchronous HTTP traffic [29]. Compared to HPA maintaining the default number of one pod, the zero-to-many scaling can reduce the cost; thus, one will pay for the time the functions are running [32]. On the other hand, it can cause a cold start problem in both HPA’s and KPA’s scaling process, as creating a new instance implies a corresponding time lag [18]. A recent study focused on understanding the relationship between the predefined settings of the default Knative auto-scaling scheme and the Quality of Service (QoS), such as workload latency [33]. To improve the efficiency of KPA, they propose the Bi-LSTM forecasting model to figure out the optimum number of pods adaptively depending on workloads. However, it only provides a preliminary evaluation with the lack of detailed workload specifications; apart from that, how KPA influences workload performance with SLO constraints is relatively underexplored compared to HPA.

## 3. Benchmarking Overview

This section describes the aim and objectives of our evaluation before demonstrating and discussing the scaling implications when utilizing FaaS applications. We first identify the relevant metrics in existing serverless auto-scaling mechanisms. Then, we carefully select a set of open-source workloads while varying the resource intensiveness and the functions’ runtime to empirically analyze the relationship between scaling-related configurations and the behavior of auto-scalers, and provide insights on how to set the configurations.

### 3.1. Aim and Scope

We aim to provide guidelines to self-hosted serverless platform providers to help them set scaling-related parameters in practice. Users developing applications as serverless functions have to make choices to properly configure metrics related to auto-scaling to be aware of workload characteristics, such as (1) how to set initial resource requests and limits when creating pods and (2) which baseline metric for HPA is appropriate for the target function. Moreover, since the platform providers should consider hardware procurement and operation costs by themselves, they should be committed to managing and provisioning containers to maximize ROI and savings. However, due to the lack of performance implications on such configurations, they might overspend their budgets due to over-provisioning resources. Furthermore, there might be room for improving the performance (e.g., tail latency) and resource utilization efficiency.

This study fills this gap, with implications for scaling FaaS workloads when utilizing state-of-the-art serverless platforms inherently dependent on K8s and Knative runtimes. For this, we conduct comprehensive measurement studies on the auto-scaling characteristics in K8s and Knative under different conditions. We choose HPA and KPA as our analysis targets, as they are default auto-scaling mechanisms provided by K8s and Knative runtime, respectively. It is worth noting that previous studies have focused on improving QoS by modifying auto-scaling schedulers and resource management policies. In contrast, we concentrate more on analyzing the efficiency of initial configurations related to auto-scaling behaviors from the viewpoint of the self-hosted serverless platform providers.

### 3.2. Custom Metrics Related to Auto-Scaling

When bootstrapping the auto-scaler in Kubernetes, an operator must specify the custom configuration YAML, which contains a condition for automatically triggering scale-out. Two core metrics affect the scaling performance and SLOs: the resource metric and the target utilization. First, the resource metric determines the type of target resource to be monitored. By default, HPA triggers a scale-out process based on the CPU, and alternative options for the base metric would be the memory or custom metrics provided by Prometheus [34]. KPA relies on metrics gathered over time-based windows, such as concurrency (by default) and request per second (rps). Once the target resource metric is determined, an operator should specify the target utilization. The target utilization is a scaling threshold specifying the boundary to decide whether the scale-out procedure is required, directly affecting the auto-scaling behavior. For example, the target utilization value followed by the CPU metric in HPA specifies the percentage of the initially allocated CPU usage limit that triggers the scaling process. When deploying FaaS-based services on top of Kubernetes, the platform providers need to make a proper decision on such metrics by considering the target workloads for efficient auto-scaling of pods.

Another main factor related to auto-scaling is the initial resource allocation of pods. Typically, commodity FaaS platforms (e.g., AWS lambda) charge the resources used based on the execution time of functions and the number of requests. Each cloud service provider has its own billing policies and resource allocation options before deploying instances (nodes) [1,3] so that clients refer to them when determining the initial resource allocation by considering their SLO requirements and budgets. The initial resource allocation influences the auto-scaling behavior with the target utilization metric. Depending on the initial resource allocation, a load generated by a workload may not reach the target utilization without creating additional replicas or incur auto-scaling when the initial allocation is insufficient. As the auto-scaling performance is tightly coupled with cloud managers’ objectives, such as the latency SLOs (e.g., response time), self-hosted FaaS platform operators who have delegated power on controlling the customer tenants, including resource management, should be aware of the initial resource allocation of pods to comply with the SLA.

### 3.3. Benchmark Suites and Workload Specification

**Figure 2:**
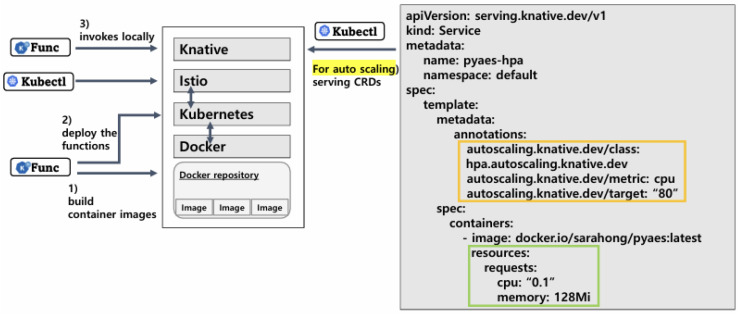
An overview of FaaS environmental setup for function deployment and invocation. (related to scaling threshold: orange, initial allocation: green).

To achieve empirical performance values reflecting various workload characteristics, we first set up a FaaS platform environment, as Figure 2 illustrates. Considering a conventional open-source FaaS platforms’ deployment model, we co-utilize Istio and Knative as runtime environments on top of Kubernetes and Docker engine [11,13]. Three main steps are required to achieve function-as-a-service on the Knative runtime: build, deploy, and invoke. We use Knative’s CLI, func, to build and push a Docker image to the image registry. Once the container image has been registered, a function can be deployed as a Knative service (ksvc). Finally, we use Istio, which provides a uniform way to connect, secure, manage, and monitor many contemporary cloud-native microservice architectures in Kubernetes [35] for the shared ingress gateway to serve all incoming traffic, and the gateway invokes the ksvc. This allows Knative to extend the container-based applications on Kubernetes using CRDs to enable a higher level of abstractions [36]. At the same time, managed service providers need to make service CRDs with YAML files to configure auto-scaling mechanisms. As the rightmost specification shows, the YAML file contains the aforementioned metrics (Section 3.2), including the initial resource allocation of the pod’s request and limit, resource metric, and target utilization of the auto-scaling mechanism’s threshold.

Then, we diversify the deployed functions sourced from the various open-source benchmark suites to stress tests provided by existing serverless platforms [37,38,39,40,41]. First, we use several functions provided by FaaSDOM [40], providing a set of benchmark tests with a variety of implementation languages compatible with major FaaS solutions [1,2,3,4] while computing price estimations. Likewise, Severlessbench offers benchmark suites for evaluating serverless platforms, such as AWS lambda, OpenWhisk, and Fn [1], which can identify the critical metrics of serverless computing. In particular, Serverlessbench aims to address several challenges in architecting a serverless application that handles both startup latency and performance isolation problems with real-world use cases. We use the matrix-multiplication workload from FaaSDOM and the alu from Serverlessbench as CPU-intensive workloads. In addition, we utilize autoscale-go, a stress test written in go for evaluating auto-scaling capabilities provided by the recent Knative release. Finally, we employ a set of workloads from the open-source benchmark suites, such as pyperformance [42] and sfc-stress [39]. The sfc-stress suite is an open-source benchmark to emulate a custom Kubernetes-ready synthetic service chain containing CPU- and memory-intensive workloads. Table 1 summarizes the detailed specifications of workloads and how we utilize them for evaluating auto-scalers. By composing the combinations of the benchmarks mentioned above, we examine the default auto-scalers in Kubernetes to determine which options would be appropriate for evaluating auto-scaling performance. Note that we also measure the standalone execution time of each function in our experimental environment.

## 4. Evaluation and Implications

To explore the performance implications on existing auto-scaling mechanisms when determining the aforementioned metrics, we evaluated various serverless benchmarks as functions on the FaaS platform environment. Our test environment consisted of a Kubernetes cluster with six nodes, a single master node, and five worker nodes using Docker 20.10.22 and Kubernetes 1.26.0 on OpenStack with an Intel(R) Xeon(R) CPU E5-2695 v4 @ 2.10 GHz (36 cores) and 512 GB of RAM. Note that such an environment is comparable to the existing studies that explore the auto-scaling behaviors of the Kubernetes cluster [16,43,44]. Each node ran on a virtual machine using Ubuntu 20.04, allocating 8 CPU cores and 16 GB RAM, respectively. On top of the Kubernetes cluster, we set up Knative v1.8.3 with Istio v1.8.1 as a networking layer. We used Hey load generator [45] while varying the duration of requests and the concurrency level to invoke functions as HTTP requests. All experiments were conducted ten times repeatedly.

Unless otherwise noted, we proceeded with our evaluation with the following conditions. We set up each pod’s initial CPU and memory allocation as 0.1 vcpu and 128 Mi (Mi denotes Mebibyte, $2^{20}$ bytes), the default configuration of existing commodity FaaS solution [46]. In the case of target utilization, we used the initial setting of HPA and KPA, 0.8 by default, for both the CPU metric and the concurrency metric. For example, HPA conducts a scaling procedure if the CPU utilization when running the target function reaches its threshold of 80 millicpus (80% of 0.1 vcpu). The value set in this way is configured as the request value of the pod, and the limit value follows the default config-deployment of Knative-serving, which is 0.2 vcpu and 1 Gi (Gi denotes Gibibyte, $2^{30}$ bytes) [47]. For example, a container can use more resources than its request for that resource specifies if the node where a pod is running has enough available resources. However, the container cannot use more than its resource limit [48]. To evaluate the QoS efficiency of workloads for each auto-scaling configuration, we measured the tail (99-percentile, 95-percentile, and 90-percentile) and median latencies, which are related to the latency-based SLOs. In addition, we tracked the number of HTTP 200 responses for each test case to examine whether it successfully handled incoming HTTP requests. Table 2 shows the overall results of the individual tests.

### 4.1. Correlation between Auto-Scaling and the Initial Resource Allocation of Pods

As discussed in Section 3.2, a decision on how to set up the initial resource allocation of pods implicitly employs different scale-out behaviors. To investigate the relationship, we explored the HPA to answer the following questions: (1) Is allocating more initial resources consequently effective to clients in terms of QoS? (2) How does the initial resource allocation of pods used as the scaling threshold affect the auto-scaling behavior? We used two CPU-intensive workloads for the experiments, the alu workload and the md5 function. We generated the load with 20 concurrent requests in a query per second for 200 s (4000 requests in total). Note that the number followed by the workload (e.g., md5-0.1-80) denotes how we configured the pod’s initial resource (0.1 vcpu) and target-utilization percentage of the CPU as a scaling threshold (80%).

**Figure 3:**
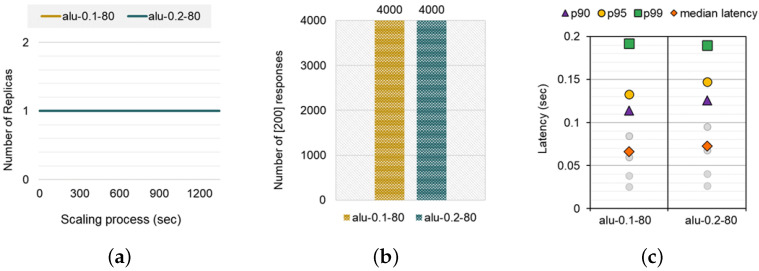
(**a**) Comparison of the scaling process with different initial pod resource settings. (**b**) Comparison of the Number of [200] responses. (**c**) Comparison of the overall latency distribution.

We first measured performance metrics related to auto-scaling with a simple alu function and 146 mcpu of the peak CPU usage, which performs arithmetic operations. Specifically, we observed three metrics: (1) the number of replicas during the entire scaling process, (2) the total number of responses received, and (3) the median and tail latencies while varying the initial resource allocation (0.1 vcpu vs. 0.2 vcpu). Figure 3 shows the results. As it did not incur a heavy workload, the number of replicas for both alu-0.1-80 and alu-0.2-80 cases were monitored as a single replica, an initial state, during the scale-out procedure, as shown in Figure 3a. Without any scale-out, HPA successfully handled the overall incoming requests (4000 out of 4000) for both alu-0.1-80 and alu-0.2-80 (Figure 3b). Finally, Figure 3c illustrates the latency distribution for both cases. As expected, the alu-0.2-80 case (0.17 s) has a 5% lower p99 latency than the alu-0.1-80 case (0.18 s), which means that allocating more initial resources improves the QoS in terms of latency SLO. We note that operators need to be aware of the additional cost required to allocate more resources to quantify the efficiency of improving QoS. Since calculating the financial expense of on-premise hosted FaaS services is not trivial and is affected by the hardware procurement and operations of the hosting entity, we mimicked the Google Cloud function calculator [46] to quantify the charge. When we calculated the fee of invoking alu functions on the commodity FaaS solution, the charge of alu-0.1-80 was USD 15.76 and alu-0.2-80 was USD 18.32, respectively. In this case, paying an additional 14% fee for improving 5% latency might not be tempting from the cloud operators’ perspective. As we demonstrated in this scenario, cloud managers can decide whether to allocate more initial resources by comparing the latency improvement and additional price increase by allocating more resources.

**Figure 4:**
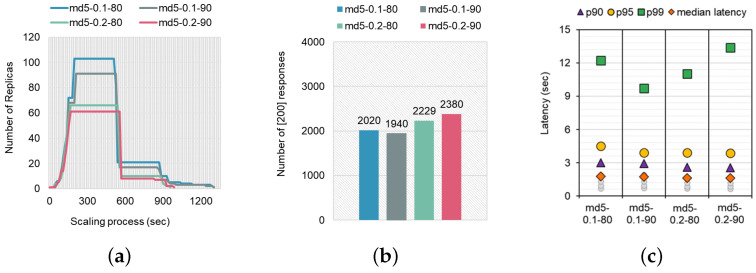
(**a**) Comparison of the scaling process with different initial pod resource settings. (**b**) Comparison of the Number of [200] responses. (**c**) Comparison of the overall latency distribution.

Similarly, we measured the aforementioned metrics for another CPU-intensive workload, the md5. It generates a number of Diffie–Hellman keys and calculates their MD5 checksums. The peak CPU usage was 11,323 mcpu, 80 times higher than the alu benchmark. Figure 4 illustrates the result. In this subsection, we mainly focus on analyzing the differences between two pairs, which have the same scaling threshold but differ in the initial CPU allocation (e.g., md5-0.1-80 and md5-0.2-80). In contrast to the alu case that does not generate additional replicas, the md5-0.1-80 case scales out up to 103 replicas and that for md5-0.2-80 is 66 replicas (Figure 4a). Meanwhile, the scaling durations were also different, the scale-in duration to one were 1305 s and 960 s, respectively. We observe that the lower the scaling threshold, the shorter the scaling duration consumes and the larger the maximum number of replicas becomes. In addition, the initial scale-out occurred in both cases in 45 s. Scaling out to the maximum number of replicas occurred 30 s faster when the initial resource allocation of pods was doubled, with the md5-0.1-80 case taking 195 s and the md5-0.2-80 case taking 165 s. The result shows that doubling the initial resource allocation of pods resulted in a faster scaling process with fewer replicas.

To quantify the efficiency of the scaling process, we define a metric for evaluating the scaling efficiency by calculating the used replicas during the entire scaling duration. The below equation illustrates the definition of scaling efficiency, where T denotes the scaling duration. For this, we sample and digitize the number of replicas and multiply them by the collection interval period (15 s) to approximately calculate the integral of the area under the line.

(1) $$\mathrm{scaling}\;\mathrm{efficiency}=\int_{0}^{T}replicas\left(x\right)dx≃\sum_{i=0}^{T}replicas\left(x_{i}\right)$$

The area of resources occupied by each case during the scaling process was 49,635 for md5-0.1-80 and 32,160 for md5-0.2-80. Allocating twice the resources consumed 64% of replicas during the scaling procedure, which means that operators can conserve resources and prevent potential contention for further pod allocation. When we calculated the fee of invoking md5 functions on the commodity cloud-offering FaaS solution, similar to the alu case, the charge of md5-0.1-80 was USD 21.17 while that of md5-0.2-80 was USD 29.22, respectively. (In order to ensure that the charges were made, both were calculated with an invocation value of responses (4000)×10,000. Commercial FaaS platforms automatically scale out after utilizing a single node’s resources. However, in our experimental environment with OpenStack and Kubernetes (k8s), we needed to pre-create nodes to enable scale-out. Due to this difference in configuration, changing the resources allocated to a node during scaling is incompatible. Since the number of nodes will ultimately increase proportionately to the scale-out quantity of pods, we assigned the resources allocated to instances to those allocated to pods). Likewise, such quantification helps self-hosted FaaS service managers to judge whether to allocate more initial resources by comparing the scaling efficiency and additional price raised by allocating more resources.

Next, we measured the performance metrics related to SLO requirements, including the number of responses and latencies. The number of responses decreased compared to the alu case, as Figure 4a shows. We observe that the md5-0.1-80 returned 2020 responses while the md5-0.2-80 returned more responses at 2229 out of 4000 requests, as shown in Figure 4b. This result implies that to successfully handle the load for the md5 workload, an additional CPU resource allocation is required. Figure 4c depicts the overall distribution of p90 tail latency to p99 tail latency and median latency. The p99 tail latency was about 12 s, and the median latency was 1.8 s in the md5-0.1-80 case, showing that md5-0.2-80 (tail: 11 s and median: 1.6 s) was effective in terms of tail latency while allowing more responses. As the result shows, allocating double the resources improves a certain level of effectiveness in clients’ QoS, but this will increase the amount charged. At the same time, additional invocations are necessary when the responses do not arrive as expected due to the lower resource allocation, which leads to additional fees. Again, the proportion of improvements in the number of successful responses and tail/median latencies would be guidelines for operators to be aware of both the SLO requirements and financial efficiency.

In summary, allocating twice the initial resources delivers better client QoS with less resource contention, known as the noisy neighbor problem [14]. However, an additional charge for doubling initial resources does not proportionally lead to performance improvements in the scaling efficiency, tail latency, and number of responses. Therefore, on-premise hosted FaaS platform operators need to establish a strategy by considering the potential quantified improvements on the metrics when configuring HPA to maximize the gain from doubling the initial resource allocation.

To intuitively compare the priority among the various scaling-related metrics, we calculate how each scaling metric affects the scaling efficiency and QoS most sensitively. As Table 3 shows, when the resource allocation ratio was set to 1:2, the degree of change due to differences in these settings was only about 0.7 times for scaling efficiency and up to 1.2 times for QoS. By comparison, when the scaling threshold was 1:1.125, the scaling efficiency and QoS showed a maximum rate of change of about 0.9 times and 1.2 times, respectively, as Table 4 summarizes. Thus, among resource allocation values and scaling thresholds, the scaling sensitivity is more affected by the scaling threshold. So, the cloud administrators can first adjust the scaling performance by changing the threshold.

### 4.2. Auto-Scaling Efficiency Depending on Scaling Threshold

To explore the correlation between clients’ QoS and the scaling threshold, we estimate performance metrics stipulated as SLA, including tail/median latencies and the number of responses [49]. For this, we compare the scaling tendency depending on the scaling threshold, which is directly related to the scaling aggressiveness of CPU-based HPA when invoking CPU-intensive workloads: the md5 and the matrix-multiplication. Specifically, we compare the two different threshold percentages of target CPU utilization, 80 (HPA’s default setting) and 90, for both workloads. As Figure 4 illustrates, we conducted more cases to investigate the effect of the difference in target utilization while varying the initial resource allocation, 0.1 vcpu and 0.2 vcpu.

We first analyze the case where the initial resource is 0.1 vcpu, md5-0.1-80 and md5-0.1-90. As Figure 4a shows, the md5-0.1-90 case processed the load for 1305 s with 91 replicas, which is 12 fewer replicas than the md5-0.1-80 case required to process the load for the same amount of time. Accordingly, the calculated scaling efficiency using Equation (1) was 49,635 for md5-0.1-80 and 43,770 for md5-0.1-90. In Figure 4b,c, the md5-cpu-stress-0.1-80 case obtained 2020 responses, while the md5-0.1-90 case obtained 1940 responses, showing a difference of 80 responses. The p99 tail latency for the md5-0.1-80 case was 12 s, while the p99 tail latency for the md5-0.1-90 case was 10 s, respectively. Note that the md5 case showed that it serves 1980 responses in contrast to the simple alu function. To successfully handle the given requests, increasing the number of replicas is necessary. However, in the case of md5-0.1-90, the scale-out was not aggressively performed compared to the md5-0.1-80 case, so the number of responses was relatively low. Instead, it delivers lower latency because fewer resources were used for scale-out and pod scheduling. Therefore, cloud operators should be aware of this trade-off between the number of responses and tail latency when setting the scaling threshold, which controls the aggressiveness of auto-scaling for increasing the number of replicas.

We also observe the tendency by changing the scaling threshold while allocating twice the resources (0.2 vcpu), denoted as md5-0.2-80 and md5-0.2-90 cases. The md5-0.2-80 case processed the load within 960 s with a maximum of 66 replicas, while the md5-0.2-90 case could process the load within 990 s with 61 replicas. The scaling efficiency was 32,160 for md5-0.2-80 and 29,865 for md5-0.2-90. In both cases, the first scale-out was performed at 45 s and the point of using the maximum number of replicas was also the same at 165 s. The first scale-in process at md5-0.2-80 was performed 15 s earlier (555 s) than md5-0.2-90, and the last scale-in of md5-0.2-80 occurred at 945 s—30 s earlier than md5-0.2-90. It is noteworthy that unlike the cases with 0.1 vcpu allocated (matrix-multiplication-0.1, md5-0.1), the md5-0.2-90 workload with a higher scaling threshold returned as many as 151 more responses than the md5-0.2-80 workload (Figure 4b). However, it showed an inefficiency of about 2.4 s regarding p99 tail latency (Figure 4c). On average, the md5 workload uses approximately 115 mcpu (0.115 vcpu) per pod per invocation, and when under load, the scaled-out replicas use a total maximum of 11,323 mcpu. Therefore, the 0.2 vcpu allocated per pod in the md5-0.2 case was enough for the workload, meaning there was only a difference of five maximum replicas used to process the load when the target utilization percentage was 80% and 90%. Also, scaling out and in occurred at similar times with similar trends. We obtained such opposite results compared to the md5-0.1 cases because the allocated amount of resources was much larger than the resources used by the pod. This result states that cloud service managers should consider both scaling threshold and initial resource allocation to improve the auto-scaling efficiency.

**Figure 5:**
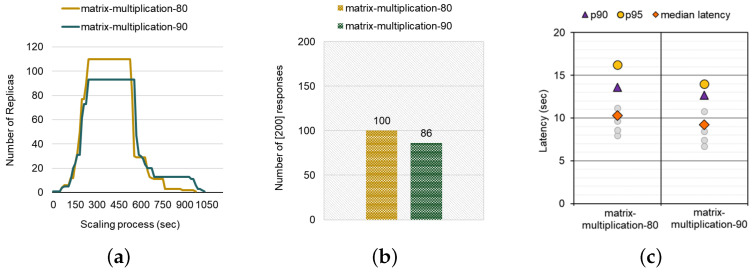
(**a**) Comparison of the scaling process with different threshold settings. (**b**) Comparison of the Number of [200] responses. (**c**) Comparison of the overall latency distribution.

Similar to the previous case study, we compare the two different threshold percentages of target utilization, 80 (default setting) and 90, for the matrix-multiplication workload (CPU utilization: 9467 mcpu in total). We generate the load by sending requests for 200 s with a concurrency level of 20 in a query per second. Note that the number followed by the workload in Figure 5 (e.g., matrix-multiplication-80) denotes how we configure the target CPU utilization as a scaling threshold. First, Figure 5a describes the number of replicas during the entire scaling procedure. The result shows that the calculated value of the matrix-multiplication-90 (43,020) is smaller than that of the matrix-multiplication-80 (44,820) workload, which means setting a higher scaling threshold is effective in conserving resources and preventing resource contention for the further warm start of existing replicas from the perspectives of on-premise hosted FaaS platform managers. Interestingly, the initial scaling-out point (45 s) and the point at which they scaled out to the maximum replicas (240 s) are the same. This result implies that the aggressiveness of scaling does not affect the scale-out point, and it is determined by the scraping period to monitor resource utilization conducted by the Metrics-server. In contrast, the scale-in to a single pod takes shorter when we scale out replicas more aggressively. Note that the HPA synchronizes resource usage collected by the Metrics-server every 15 s to adjust the scaling. This trend is because the replicas took longer to scale-in since the CPU usage per pod was higher, as there were fewer replicas in the matrix-multiplication-90 case compared to the matrix-multiplication-80 case.

Next, we measure the performance metrics related to SLO requirements, including the number of responses and latencies. Figure 5b shows that the matrix-multiplication-80 workload had 100 responses while the matrix-multiplication-90 had 86 responses, indicating that the lower scaling threshold handles the load 16% better. If the number of responses does not meet the client’s requirements, it might lead to additional invocations of the function to fulfill the desired rate. We note that serverless billing in the commercial FaaS platforms is typically affected by three factors: the initial resource allocation of pods, the execution time of function, and the number of requests. This means that dropping the scaling threshold would be cost-effective for FaaS clients.

In contrast, the metrics related to latency-SLO have an opposite tendency, similar to the md5-0.1 cases. Figure 5c shows the estimated latency distribution for both cases. The tail latency (95 percentile) for the matrix-multiplication-80 (16 s) is 14% slower than the matrix-multiplication-90 case (14 s). Again, we observe a similar trend to the md5-0.1-80 vs. 90 cases with a trade-off between the number of responses and the latency. The matrix-multiplication-90 workload better satisfies the SLA in terms of latency while sacrificing the number of responses.

In summary, there are two possible strategies for operators to conserve resources and prevent resource contention: (1) to set a higher target-utilization-percentage and scale slowly to use fewer replicas; (2) to set a lower target-utilization-percentage and scale aggressively to use more replicas but finish the scaling process quickly.

### 4.3. Auto-Scaling Behavior of HPA Depending on Base Resource Metrics

To investigate the impact of base metrics on auto-scaling behavior, we trace the number of replicas and examine the number of responses and the latencies according to the scaling duration when utilizing HPA. We ask ourselves two fundamental questions: (1) What is the proper scaling threshold when using memory as a base metric for HPA? (2) Is the memory metric actually effective for typical memory-intensive workloads compared to the default base metric (CPU)? For comparison, we generate a request to run the autoscale-go (memory-intensive) workload under two different base metric conditions, CPU and memory. We create a load configured as 30 s of duration to send requests with a concurrency level of 50 (queries per second).

We first conduct a preliminary experiment to figure out the inter-relationship between the initial memory allocation of the pod and memory-based HPA depending on the working set size of workloads. In contrast to CPU-based HPA, which monitors the percentage of CPU utilization (0.8 by default), the threshold is specified as a constant value. However, there is no recommendation or given examples when scaling on the memory metric with open-source FaaS solutions, meaning that FaaS clients need to determine the value according to workloads. Followed by Google Cloud Function’s minimum request, which is 100 mcpu and 128 Mi, we proportionally set the threshold as 102 Mi by multiplying 0.8 by the initial memory allocation (128 Mi). In this case, we observe that HPA continuously creates unusable replicas even with deploying workloads only before generating load. This is due to the lack of memory resources, as both workloads’ initial working set size is approximately 160 Mi. This result indicates that the allocated threshold should be at least as large as the working set when using memory-based HPA for workloads.

**Figure 6:**
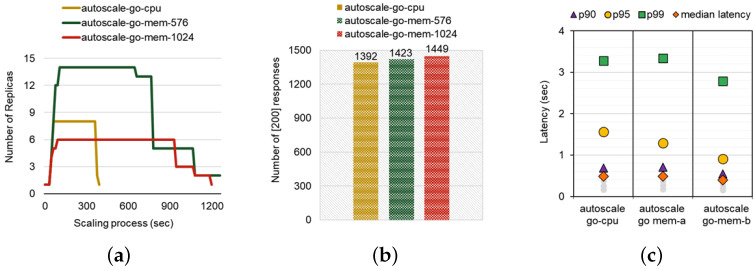
(**a**) Comparison of the scaling process. (**b**) Comparison of the Number of [200] responses. (**c**) Comparison of the overall latency distribution.

Figure 6 represents the scaling behavior of the autoscale-go workload when applying CPU-based HPA and memory-based HPA. The peak resource utilization of the autoscale-go workload was estimated as 942 mcpu and 7289 Mi, respectively. To avoid the aforementioned inefficient auto-scaling situation, we increase the target threshold of memory-based HPA to 576 Mi and 1024 Mi, respectively. Considering the initial working set size of the autoscale-go workload (160 Mi), we empirically derive the threshold of 576 Mi (160 Mi × 4.5 × 0.8) from mimicking the CPU-based HPA’s threshold of 80%. We first focus on comparing the mem-576 Mi and the mem-1024 Mi cases. Based on the result, increasing the threshold reduces the maximum number of replicas during the scaling procedure. The mem-1024 Mi case with a 1.7× higher threshold increases the replicas up to six, while the maximum number of replicas for mem-576 Mi is estimated at 14 (2.3×). This result states that cloud service managers would be preferred to raise the scaling threshold as it reduces the maximum replicas in favor of the ratio.

Compared to CPU-based HPA, both the initial scaling-out point and the point that reaches the maximum replicas are slower in memory-based HPA cases. Each of the three workloads took 60 (CPU), 105 (mem-576 Mi), and 90 (mem-1024 Mi) seconds until the maximum replicas, respectively. This result implies that CPU-based HPA is more reactive than memory-based HPA when requiring the scale-out procedure, even for memory-intensive workloads. Moreover, the entire scaling procedure of the CPU-metric case until the scale-in to a single pod is faster than that for both memory-metric cases. This is due to the nature of memory allocation of Kubernetes-based systems. It cannot be freed until it is determined to be ‘unused’ and continues to be occupied until garbage collection occurs, resulting in a longer scaling duration. It might lead to resource waste as the replicas that have been scaled out are maintained without going through the scale-in process. Meanwhile, the memory-based HPA requires fewer replicas to control the load than the CPU-based HPA when increasing the memory threshold size up to 1024 Mi. But still, the scaling process was lengthy because of its larger memory footprint. Accordingly, the calculated scaling efficiency of the CPU-metric case (2655) is smaller than the mem-576 Mi (12,105) and mem-1024 Mi (6030). This result states that utilizing the CPU-metric is even effective for memory-intensive workloads in terms of scaling efficiency. Therefore, operators must carefully select the default metric considering CPU and memory utilization simultaneously to reduce the scale-in period.

Regarding the performance results shown in Figure 5b,c, the memory-based HPA delivers better performance than the CPU-based HPA regarding SLO requirements. As the trigger metric, memory-based threshold was set higher, the number of responses increased, and the p99 tail latency, p95 tail latency, and median latency also became shorter. HPA handles 1392, 1423, and 1449 responses for 1500 incoming requests for CPU-based HPA and Memory-based HPA with 576 Mi and 1024 Mi, respectively (Figure 5b). Memory-based HPA with a higher threshold (mem-1024 Mi) obtained 4% and 2% more responses than mem-576 Mi and CPU-based HPA. Furthermore, Figure 5c illustrates the latency distribution for both cases. The autoscale-go-mem-1024 case (2.7908 s) has a 16% and 15% lower p99 tail latency than the autoscale-go-mem-576 case (3.3401 s) and autoscale-go-cpu case (3.2778 s), which means that setting a higher threshold in Memory-based HPA improves the QoS in terms of latency SLO. In this case, using CPU-metric for saving 56% scaling efficiency, for giving up 15% latency, or for improving 2% number of responses might be attractive from a manager’s perspective.

## 5. Selecting a Default Auto-Scaling Policy: HPA vs. KPA

In this section, we conduct a case study to investigate the auto-scaling tendency when utilizing HPA and KPA depending on the concurrency level of the given load. Recent commoditized Kubernetes-based systems [50,51] often co-utilize Knative and Istio runtimes on top of Kubernetes, which becomes one of the standard ways of establishing an infrastructure for FaaS platforms. Such a deployment model allows cloud operators to choose a default auto-scaling mechanism, a traditional Kubernetes-based HPA, and KPA provided by the Knative runtime. As explained in the previous section (Section 2), HPA and KPA use different base metrics for scaling, CPU utilization (or memory) and concurrency, respectively. However, it is still unclear which auto-scaling policy is effective due to the lack of guidelines or evaluation studies. Therefore, we try to figure out the answer to the following questions: Which default auto-scaler is advantageous to use depending on the concurrent level of the load? Would the KPA that designates concurrency as the base metric handle concurrent loads better and satisfy clients’ QoS more? To figure this out, we invoke the pyaes function, a CPU-intensive workload that performs AES encryption while varying the level of concurrent load from 1 to 600. We configure a load with 30 s of duration to send requests in queries per second. We keep the HPA and KPA targets at the default values specified in Knative for a fair comparison. The HPA was set to 80 mcpu, while the KPA had a target concurrency of 100 and a target-utilization percentage of 80%, with a threshold of 80.

**Figure 7:**
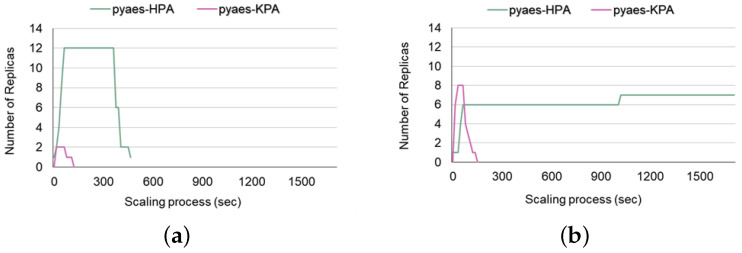
(**a**) Concurrency level of 80. (**b**) Concurrency level of 600.

**Figure 8:**
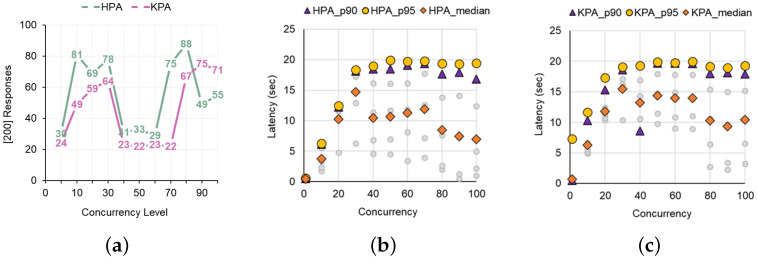
(**a**) Trends in response according to concurrency level (1 to 100). (**b**) Latency distribution trends of HPA. (**c**) Latency distribution trends of KPA.

**Figure 9:**
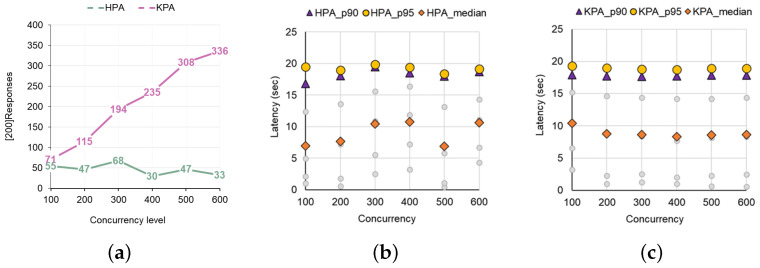
(**a**) Trends in response according to concurrency level (100 to 600). (**b**) Trends in SLA of HPA. (**c**) Trends in SLA of KPA.

Next, we estimate the SLO-related values for KPA and HPA while increasing the concurrency level of the loads from 1 to 600 in Figure 7. To precisely figure out the cross point where utilizing KPA is efficient, we assess the number of responses and latencies more fine-grained from concurrency levels 1 to 100. According to Figure 8, which presents the number of responses and SLA latency distribution when varying the concurrency level from 1 to 100, using the HPA auto-scaler generally resulted in more responses and was more effective in terms of SLA latency than using KPA auto-scaler (Figure 8b,c). However, as the concurrency level exceeded 80, these results were reversed. Figure 9a,b show the results of the experiment with various concurrency levels from 100 to 600. As shown in Figure 9a, from the point where the concurrency level exceeds the threshold (100×0.8) set up in KPA, it shows a generally more effective trend than HPA in terms of the number of [200] responses and in terms of the p95 tail latency and median latency shown in Figure 9b.

To summarize, when performing load testing by changing the concurrency level for a single workload, the default HPA and KPA in Knative showed that if the concurrency level does not exceed the KPA’s target utilization (100×0.8) that has been pre-set, then, in general, KPA was more effective than HPA in terms of the number of responses and SLA latency. However, when it exceeds the boundary, KPA returns more responses and has a much shorter latency to handle the load than HPA. Ultimately, the effective range when using both workloads varies depending on how the concurrency level of the workload and the scaling threshold of HPA and KPA are set. The results of this experiment give lessons on which auto-scaler is advantageous for private cloud owners, depending on the concurrency level of the load.

## 6. Conclusions

This research paper examined the implications of employing Kubernetes and Knative, two widely recognized container orchestration solutions, to facilitate the self-hosted FaaS platform run in on-premise. In particular, we thoroughly explored the impact of configurations, such as the type of metrics, thresholds, and initial pod resources, related to the state-of-the-art automatic scaling mechanism, HPA. In addition, we conduct a comparison study between HPA and KPA for a typical CPU-intensive workload, analyzing their behaviors when subjected to different concurrency levels during load testing in Knative.

- We obtained the result that since scaling is aggressive due to the lower target-utilization percentage, it consumes energy to create replicas and, as a result, returns a small number of responses. However, having many replicas produced in this way ensures high efficiency in terms of latency. Through the discovered logic, resource allocation for the pod should be performed after determining whether the QoS focuses on the number of responses or SLA latency.
- We figured out that assigning more resources to the pod does not always shorten the latency SLO. It states that cloud service managers should consider both the scaling threshold and initial resource allocation to improve the auto-scaling efficiency.
- Unconditional memory-based scaling with memory-intensive workloads is not beneficial; one can choose whether to use memory-based HPA to meet QoS or CPU-based HPA to ensure scaling efficiency.
- When running FaaS with different load levels in Knative, it is only beneficial from a QoS standpoint to use KPA, which uses concurrency as the default scaling metric. Autoscaler should be selected after identifying the scaling threshold and the load concurrency level applied to the function. Based on the KPA’s default target threshold, the use of HPA when dealing with simultaneous levels of load less than the KPA’s scaling threshold may be a way to satisfy the QoS of the client further.

The findings of this paper serve as a guideline for users who seek to deploy functions in on-premise hosted FaaS platforms or help them meet SLO requirements by improving the QoS.

The paper has some limitations:

- **Threats to validity in precise QoS comparison regarding cost-effectiveness:** Since the cost for on-premise hosted software could not be calculated correctly due to diverse considerations (e.g., hardware procurement and operations), comparing cost parts relevant to QoS is non-trivial. We followed Google Cloud Function’s cost procedure as it fits our initial resources (e.g., CPU and memory) for the pods we set up. However, due to the difference between self-hosted platforms running on-premise and public cloud, an accurate cost assessment should be reviewed to determine whether QoS benefits can be obtained.
- **Experiments on a limited FaaS platform, only Knative:** Among the various Kubernetes-based self-hosted FaaS platforms, all the experiments on the paper were conducted only in the Knative environment. Therefore, evaluating other self-hosted FaaS platforms is necessary before integrating with our scaling methodology.

Future endeavors include expanding the experiments to encompass more scenarios and developing an adaptive scaling mechanism for various workloads regarding limitations.

## Figures and Tables

**Table 1:** Detailed specification of deployed functions.

Function	Type	Runtime	Standalone Execution Time (s)	Evaluation Purpose
alu [38]	CPU-intensive	Python	0.32	initial resource allocation (Section 4.1)
md5 [39]	CPU-intensive	Node.js	0.97	initial resource allocation (Section 4.1)
matrix-multiplication [40]	CPU-intensive	Python	5.42	scaling threshold (Section 4.2)
autoscale-go [41]	Memory-intensive	Go	0.12	base metric (Section 4.3)
pyaes [37]	CPU-intensive	Python	0.39	HPA vs KPA (Section 5)

**Table 2:** Overall evaluation specification.

	Scaling Efficiency	QoS (Responses)	QoS (Latency SLO)
alu-0.1-80	-	4000	0.17 s
alu-0.1-90	-	4000	0.18 s
md5-0.1-80	49,635	2020	12 s
md5-0.2-80	32,160	2229	11 s
md5-0.1-90	43,770	1940	10 s
md5-0.2-90	29,865	2380	13.4 s
matrix-multiplication-80	44,820	100	16 s
matrix-multiplication-90	43,020	86	14 s
autoscale-go-cpu	2655	1392	3.2778 s
autoscale-go-mem-576	12,105	1423	3.3401 s
autoscale-go-mem-1024	6030	1392	2.7908 s

**Table 3:** Relative comparison to quantify how the ratio of resource allocation sensitively affects the performance.

	md5-0.1-80	md5-0.2-80	md5-0.1-90	md5-0.2-90
scaling efficiency	1	0.648	1	0.682
responses	1	1.103	1	1.227
latency SLO (s)	1	0.917	1	1.134

**Table 4:** Relative comparison to quantify how the scaling threshold sensitively affects the performance.

	md5-0.1-80	md5-0.1-90	md5-0.2-80	md5-0.2-90
scaling efficiency	1	0.882	1	0.929
responses	1	0.960	1	1.07
latency SLO	1	0.833	1	1.218

## Abbreviations

The following abbreviations are used in this manuscript:

FaaS	Function-as-a-Service
k8s	Kubernetes
SLO	service level objective
SLA	service level agreement
QoS	quality of service
CSP	cloud service providers
HPA	horizontal pod auto-scaler
VPA	vertical pod auto-scaler
KPA	Knative pod auto-scaler
rps	request per second
